# Relative protein intake and associations with markers of physical function in those with type 2 diabetes

**DOI:** 10.1111/dme.14851

**Published:** 2022-04-21

**Authors:** Joseph Henson, Frank Arsenyadis, Emma Redman, Emer M. Brady, Nicole A. Coull, Charlotte L. Edwardson, Andrew P. Hall, Lewis J. James, Kamlesh Khunti, Alex V. Rowlands, Emma J. Stevenson, Daniel J. West, Melanie J. Davies, Thomas Yates

**Affiliations:** ^1^ NIHR Leicester Biomedical Research Centre Leicester UK; ^2^ Diabetes Research Centre College of Life Sciences University of Leicester Leicester UK; ^3^ 4490 University Hospitals of Leicester NHS Trust Leicester UK; ^4^ Department of Cardiovascular Sciences University of Leicester Leicester UK; ^5^ Department of Health Sciences University of Leicester Leicester UK; ^6^ Hanning Sleep Laboratory Leicester General Hospital Leicester UK; ^7^ School of Sport, Exercise and Health Sciences Loughborough University Loughborough UK; ^8^ NIHR Applied Health Research Collaboration – East Midlands (NIHR ARC‐EM) Leicester Diabetes Centre Leicester UK; ^9^ Alliance for Research in Exercise, Nutrition and Activity (ARENA) Sansom Institute for Health Research Division of Health Sciences University of South Australia Adelaide Australia; ^10^ Population Health Sciences Institute Newcastle University Newcastle upon Tyne UK

**Keywords:** frailty, physical function, protein intake, reference nutrient intake, type 2 diabetes

## Abstract

**Aims:**

To examine the independent associations between relative protein intake (g kg^−1^ day ^1^) and markers of physical function in those with type 2 diabetes, while also comparing with current guidelines for protein intake.

**Methods:**

This analysis reports data from the ongoing Chronotype of Patients with Type 2 Diabetes and Effect on Glycaemic Control (CODEC) study. Functional assessments included: Short Physical Performance Battery (SPPB), 60 s sit‐to‐stand (STS‐60), 4‐m gait speed, time to rise from a chair (×5) and handgrip strength. Participants also completed a self‐reported 4 day diet diary. Regression analyses assessed whether relative protein intake was associated with markers of physical function. Interaction terms assessed whether the associations were modified by sex, age, HbA1c or body mass index (BMI).

**Results:**

413 participants were included (mean ± SD:age = 65.0 ± 7.7 years, 33% female, BMI = 30.6 ± 5.1 kg/m^2^). The average total protein intake was 0.88 ± 0.31 g kg^−1^ day^−1^. 33% of individuals failed to meet the reference nutrient intake for the United Kingdom (≥0.75 g kg^−1^ day^−1^), and 87% for European recommendations (≥1.2 g kg^−1^ day^−1^). After adjustment, each 0.5 g/kg of protein intake was associated with an 18.9% (95% CI: 2.3, 35.5) higher SPPB score, 22.7% (1.1, 44.3) more repetitions in STS‐60, 21.1% (4.5, 37.7) faster gait speed and 33.2% (16.9, 49.5) lower chair rise time. There were no associations with handgrip strength or any interactions.

**Conclusions:**

Relative protein intake was positively associated with physical function outcomes, even after consideration of total energy intake. As a number of individuals were below the current guidelines, protein intake may be a modifiable factor of importance for people with type 2 diabetes.

**Novelty statement**

**What is already known?**

- Type 2 diabetes reflects a powerful physiological model of accelerated biological ageing.
- Inadequate dietary protein has also been linked to a progressive loss of muscle function.
- Population‐wide recommendations for protein intake may not necessarily reflect the altered requirements for those with type 2 diabetes.

**What has this study found?**

- Relative protein intake was independently associated with physical function outcomes. Results were consistent across age, sex, HbA1c and BMI categories.
- 33% failed to meet the UK reference nutrient intake for protein and 87% did not reach European thresholds.

**What are the implications of the study?**

- Protein intake may be a modifiable factor of importance for people with type 2 diabetes.

## 1 INTRODUCTION

Type 2 diabetes mellitus reflects a powerful physiological model of accelerated biological ageing that influences whole body health and function.^1^ Sarcopenia and frailty risk is greater in individuals with type 2 diabetes via impaired balance, reduced flexibility, decreased relative muscle mass and muscle quality compared with healthy age‐matched controls.^2^ This reduces quality of life and the ability to perform activities of daily living.^3^

Observational studies have documented that frailty prevalence in older adults with type 2 diabetes (≥65 years) ranges from 32% to 48%, compared with 5–10% in those without type 2 diabetes.^4^ In our previous work, we found that ~30% of 635 adults (median age 66 years, BMI = 31 kg/m^2^) with type 2 diabetes have impaired physical function, with their ability to carry out functional tasks of daily living similar to those without diabetes who are over a decade older.^5^ As type 2 diabetes is a risk factor for the development of both frailty and sarcopenia, international expert consensus statements now recommend routine physical function assessments and reiterate the need for interventions to prevent diabetes‐related disabling outcomes.^2^, ^6^

Dietary protein is important for physical function, particularly in older adults and requirements vary by sex.^7^ Inadequate dietary protein, especially when accompanied with low physical activity and/or sedentary behaviour, has been linked to progressive loss of muscle mass leading to loss of muscle function, low muscle strength and slower walking speed.^8^ The UK adult reference nutrient intake (RNI) for protein which is assumed adequate for the majority of the population is 0.75 g kg^−1^ day^−1^.^9^ In comparison, European guidelines recommend a minimum of 1.2 g kg^−1^ day^−1^ for older adults with chronic conditions.^10^ These population‐wide recommendations may not necessarily reflect the altered requirements for older adults and those with type 2 diabetes, which is important given their vulnerability to functional decline and mobility at an earlier age.

Little is known about the associations between protein intake and markers of physical function, independent of physical activity in those with type 2 diabetes. Therefore, our aim was to examine the independent associations between relative protein intake and markers of physical function in those with type 2 diabetes. Our secondary aim was to compare the protein intake of those with type 2 diabetes to current recommendations.

## 2 METHODS

Participants included in this nested study had data collected at a single visit as part of the ongoing, multisite observational study (Chronotype of Patients with Type 2 Diabetes and Effect on Glycaemic Control (CODEC)) between 2016–2021^11^ (clinicaltrials.gov:NCT02973412). Briefly, participants had established type 2 diabetes for more than 6 months, an HbA1c ≤86 mmol/mol (10%) and were aged between 18–75 years. Details of other inclusion and exclusion criteria can be found in Electronic Supplementary Material (ESM) (Table 1). Ethical approval was obtained from the West Midlands‐ Black Country Research Ethics Committee (16/WM/0457). All participants gave informed written consent and were recruited from primary and specialist health care settings in Leicester, Nottingham, Derby and Lincoln, UK.

**TABLE 1:** Participant characteristics for included participants and when stratified by sex

	Included participants (*n *= 413)	Female (*n *= 137)	Male (*n *= 276)
*Demographic variables*			
Age (years)	65 ± 7.7	64.9 ± 8.0	65.4 ± 7.6
Ethnicity (white European)	380 [92.0]	130 [94.9]	250 [90.6]
Current smokers	18 [4.4]	5 [3.6]	13 [4.7]
Index of multiple deprivation rank	19828.6 ± 8973.5	21002.9 ± 9099.4	19305.5 ± 8871.6
*Anthropometric variables*			
BMI (kg/m^2^)	30.6 ± 5.1	30.4 ± 5.0	30.6 ± 5.0
Weight (kg)	89.4 ± 19.0	79.5 ± 16.6	94.3 ± 18.3
*Cardio‐metabolic variables*			
HbA1c (mmol/mol)	54 ± 4	54 ± 4	54 ± 4
HbA1c (%)	6.9 ± 0.4	6.9 ± 0.3	6.9 ± 0.5
Duration of type 2 diabetes (years)	10.8 ± 7.4	10.3 ± 7.5	11.0 ± 7.3
*Medication*			
Biguanide	264 [63.9]	83 [60.6]	181 [65.6]
Sulphonylurea	72 [17.4]	21 [15.3]	51 [18.5]
GLP−1RA	15 [3.6]	4 [2.9]	11 [4.0]
Insulin	75 [18.2]	22 [16.1]	53 [19.2]
DPP−4i	58 [14.0]	21 [15.3]	37 [4.7]
SGLT2i	32 [7.7]	13 [9.5]	19 [6.9]
Thiazolidinedione	6 [1.5]	1 [0.7]	5 [1.8]
*Device measured physical activity*			
Number of days	6.9 ± 0.3	6.9 ± 0.4	6.9 ± 0.4
Total physical activity (mg)^a^	21.6 ± 7.1	21.2 ± 5.8	21.8 ± 7.6
*Physical function*			
SPPB	11 (10, 12)	11 (9, 12)	11 (10, 12)
Number achieving an SPPB score of <10	131 [31.7]	55 [40.1]	76 [27.5]
STS−60	22.6 ± 7.0	21.5 ± 6.2	23.2 ± 7.2
4mGS (m/s)	1.0 ± 0.2	0.9 ± 0.2	1.0 ± 0.3
5STS	14.2 ± 5.7	14.8 ± 5.9	13.8 ± 5.6
Handgrip strength	30.3 ± 10.9	21.0 ± 9.7	34.8 ± 11.3
*Dietary variables*			
Total energy intake (kcal)	1622 ± 463	1485 ± 383	1689 ± 486
Carbohydrate (g/day)	186.9 ± 71.4	169.7 ± 66.7	195.1 ± 74.3
Carbohydrate (%)	46.1 ± 7.2	45.7 ± 7.0	46.2 ± 7.3
Fat (g/day)	63.6 ± 23.9	58.5 ± 19.3	66.2 ± 25.5
Fat (%)	35.3 ± 6.8	35.4 ± 7.1	35.3 ± 6.7
Protein (g/day)	75.6 ± 22.5	70.3 ± 18.2	78.3 ± 23.9
Protein (%)	18.6 ± 4.6	18.9 ± 4.5	18.5 ± 4.6
Not meeting current UK recommendations for protein intake (<0.75 g kg^−1^ day^−1^)	139 [33.7]	40 [29.2]	99 [35.9]
Not meeting current European recommendations for protein intake (<1.2 g kg^−1^ day^−1^)	360 [87.2]	111 [81.0]	249 [90.2]
Protein (g kg^−1^ day^−1^)	0.88 ± 0.31	0.93 ± 0.36	0.85 ± 0.28

Note

Data presented as median (interquartile range), number [column percentage] or mean ± SD.

Abbreviations: 4mGS, 4 m gait speed; DPP‐4i, Dipeptidyl peptidase‐4 inhibitor; GLP‐1RA, Glucagon‐like peptide‐1 receptor agonist; SGLT2i, Sodium‐glucose transport protein 2 inhibitor; SPPB, Short Physical Performance Battery; STS, sit to stand.

^a^ This value represents ENMO (Euclidean norm minus 1g).^18^

### 2.1 Demographic, anthropometric and cardio‐metabolic measures

Outcome measures were recorded by an appropriately trained member of the study team and included: age, sex (male/female), ethnicity (self‐reported and categorised as (white European, South Asian or other), duration of type 2 diabetes (years), number of type 2 diabetes medications, smoking status (current/ex/never) and body mass index (BMI) (kg/m^2^). BMI was calculated to the nearest 0.1 kg/m^2^. HbA1c was quantified using the Bio‐Rad Variant II HPLC system (Bio‐Rad Clinical Diagnostics, Hemel Hempstead, UK).

### 2.2 Dietary intake

A self‐administered 3 or 4‐day diet diary was used to assess intake. Estimated food diaries have been used successfully in many large‐scale studies, particularly where detailed food and/or nutrient intakes are required at an individual level.^12^, ^13^ Participants were given written and verbal guidance on how to complete the diet diary. Diet data were then entered into Nutritics^©^ (https://en‐gb.nutritics.com/p/home) by trained individuals following standardised operating procedures and analysed for macronutrient composition (g/d) and energy intake (kcal/d). Diet diaries that did not meet quality criteria, defined as including at least two weekdays and one weekend day with complete data, were excluded from the analysis.

### 2.3 Physical function measures

Participants completed The Short Physical Performance Battery (SPPB), which is a series of 3 tests to assess lower extremity physical function: a 4 m gait speed test (4 mGS) at usual walking pace, time to complete 5 unassisted chair stands (5STS), and 3 standing balance tests, each held for 10 s.^14^ Each test is scored on a 0 to 4 scale using previously validated norms and summed for an overall score range of 0 to 12, with 0 indicating the lowest physical performance. Of these, the 4mGS is the best characterised and could arguably be used as a single surrogate measure for physical function because it correlates highly with exercise capacity and health status.^14^ Similarly, the 5STS is closely associated with lower limb muscle strength and correlates with the incremental shuttle walk test in those with chronic disease.^15^ Given the ceiling effect associated with the balance tests (84.5% of included participants scored a maximum of 4 points) and to be consistent with previous observational work, we also report individual SPPB sub‐test results for 4mGS and 5STS.^16^

The 60 s sit‐to‐stand test (STS‐60), a strong predictor of mobility, was also used to assess muscular endurance and physical ability. Participants were asked to stand from, and return to, a standardised sitting position as many times as possible in 60 s without using their arms for support (arms placed across the chest).

Hand grip strength was assessed through the use of a handheld dynamometer. Participants were sitting while the elbow of their arm holding the dynamometer was placed against the side of their body and bent to a 90° angle with the forearm placed on an armrest. Hand grip strength was measured three times on each side, with the highest value (from either hand) taken as their maximum grip strength.

### 2.4 Index of multiple deprivation

Social deprivation was determined by assigning an index of multiple deprivation (IMD) rank to the participant's resident area (based on postcode). IMD scores are publically available continuous measures of compound social and material deprivation linked to health outcomes (including; income, employment, education, living environment and health).^17^

### 2.5 Physical activity

Participants were asked to wear an accelerometer (GENEActiv, ActivInsights Ltd, Kimbolton, UK) on their non‐dominant wrist 24 h/day for 7 days to quantify physical activity. Average acceleration in m*g* was used as a proxy for overall physical activity. The GENEActiv was initialised to collect data at 100 Hz. The device was fitted on the day of their appointment and participants returned the device at the end of the assessment period.

### 2.6 Accelerometer data processing

Data were downloaded using GENEActiv PC software version 3.2. The GENEActiv.bin files were processed using R‐package GGIR version 1.8–1 (http://cran.r‐project.org).^18^

Calculation of the average magnitude of dynamic acceleration was expressed in milligravitational units (mg). Participants were excluded if their accelerometer files showed post‐calibration error >0.01 g (10 mg), fewer than 3 days of valid wear (defined as >16 h per day^19^; or wear data was not present for each 15 min period of the 24 h cycle.

### 2.7 Statistical analysis

Demographic, anthropometric, physical function, diet, physical activity variables are presented as numbers (mean ± SD if parametric, median (interquartile range (IQR) if not non‐parametric)) or percentages for categorical groups.

Generalised linear models were used to assess whether protein intake (reported as grams of protein per kilogram of body weight (g kg^−1^ day^−1^)) was associated with markers of physical function. All models were adjusted for: age (continuous), sex, ethnicity (white European, South Asian or other), IMD (continuous), smoking status (current/ex/never), duration of type 2 diabetes, HbA1c, number of type 2 diabetes medications, overall physical activity (continuous), accelerometer wear time (continuous) and total energy intake (continuous). To minimise the likelihood of multicollinearity, we removed protein intake (kcal derived from protein = 4 kcal/g protein) from the calculation of total energy intake.

The SPPB score was analysed using Gamma regression with a log‐link, and the STS‐60 was analysed using Negative Binomial regression with a log‐link. The 4mGS, 5STS and hand grip strength data were analysed using a linear regression model with a log‐link to allow the strength of association to be compared with the SPPB and STS‐60. All models were checked for multicollinearity by examining the correlations between independent variables in the fully adjusted models. For ease of interpretation, data were back transformed to show the fold change (95% CI) in measures of physical function per 0.5 g/kg difference in protein intake.

We also sought to examine the extent to which BMI may attenuate reported associations with an additional sensitivity analysis. Models included the same covariates as listed above (plus BMI), with the exception of the exposure variable, which was reported as absolute protein intake (g/day) in order to circumvent the issue of multicollinearity exhibited between relative protein intake (g kg^−1^ day^−1^) and BMI (both derived using the same denominator (weight in kg)).

To facilitate interpretation of the findings and to confirm linearity, relative protein intake was also examined as quartiles. The lowest quartile was defined as having the lowest relative protein intake. Violin plots (incorporating density curves) were used to demonstrate the distributions, where the width of each curve corresponds with the approximate frequency of data points within each quartile. These results are also reported as estimated marginal means and *p* for trend values (assessed using polynomial contrasts), while also being adjusted for the same covariates listed above.

Interaction terms were simultaneously entered into the same model to assess whether the associations between protein intake and markers of physical function were consistent across sexes, age, HbA1c and BMI. All data were analysed using SPSS (version 26.0). A *p* < 0.05 was considered statistically significant for the main effect and interaction analyses.

## 3 RESULTS

There were 413 participants with demographic, anthropometric, accelerometer and diet data (age = 65 ± 7.7 years, 33% female, BMI = 30.6 ± 5.1 kg/m^2^). Of these, all participants had at least 3 days of complete diet data, with 33% of participants having 4 days. Table 1 displays the characteristics of all included participants, stratified by sex. ESM Table 2, also displays the characteristics of all included participants and those not included. Our sample was representative of the wider CODEC cohort except for being older (65 vs. 62.9 years), having a larger proportion of White Europeans (92.0% vs. 77.3%) and fewer people taking metformin (63.9% vs. 69.1%, GLP‐1RA (3.6% vs. 6.8%) or insulin (18.2% vs. 26.7%).

Mean energy intake was 1622 ± 463 kcal/day (including protein). Average protein consumption was 75.6 ± 22.5 g/day, corresponding to 18.6% of total energy intake and 0.88 ± 0.31 g kg^−1^ day^−1^. The minimum protein intake reported was 0.13 g kg^−1^ day^−1^, and the maximum was 1.99 g kg^−1^ day^−1^. In addition, 33.7% had protein intake ≤0.75 g kg^−1^ day^−1^, and 12.8% consumed ≥1.2 g kg^−1^ day^−1^.

**FIGURE 1:**
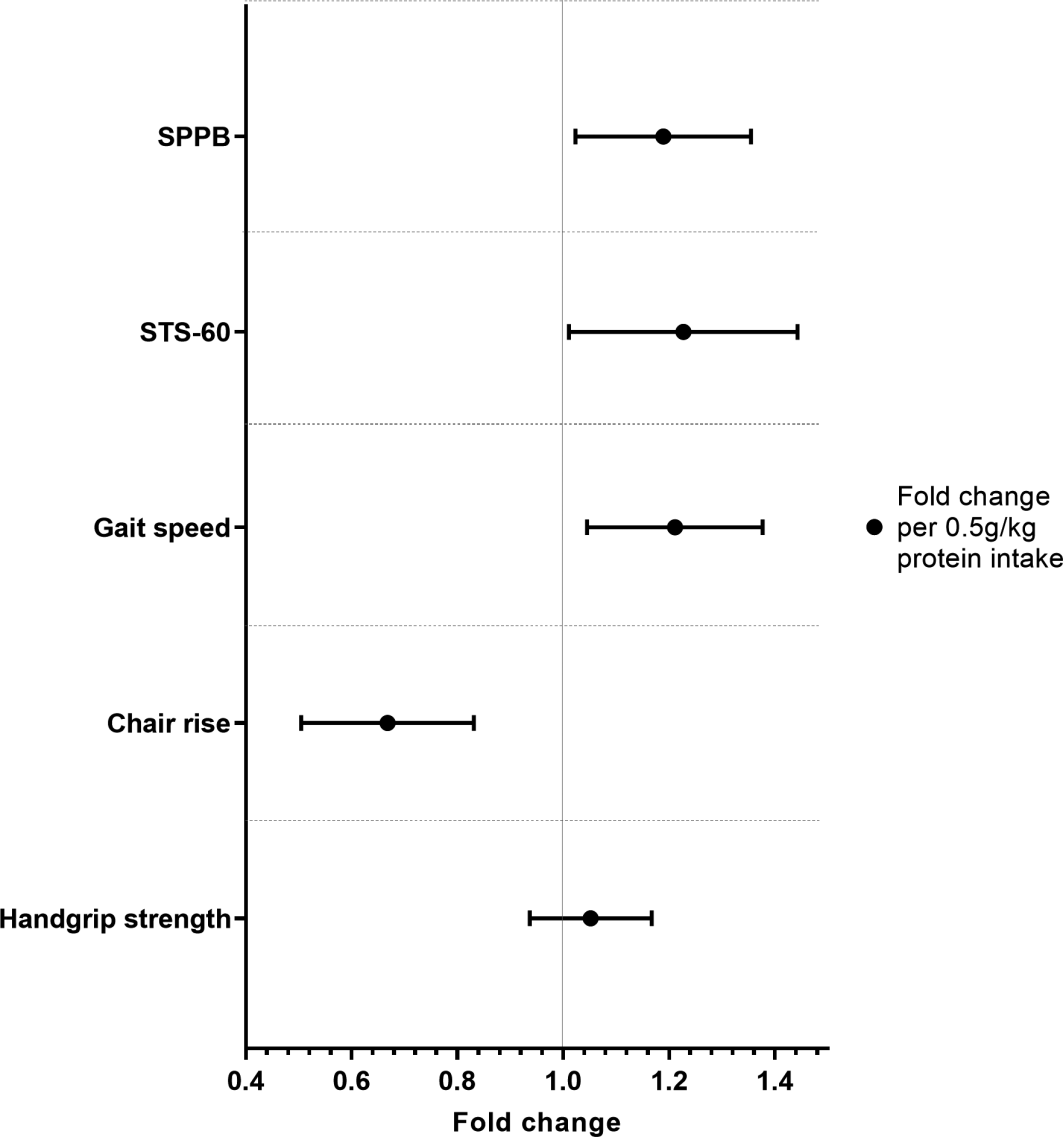
Associations between relative protein intake and functional assessments. Values represent fold change and 95% CI for each 0.5 g/kg change in protein intake. Adjusted for age, sex, ethnicity, index of multiple deprivation, smoking status, duration of type 2 diabetes, HbA1c, number of type 2 diabetes medications, overall physical activity, accelerometer wear time and total energy intake (minus protein)

The associations between protein intake and markers of physical function are shown in Figure 1 (data also shown in Electronic Supplementary Material, Table S3). After adjustment for age, sex, ethnicity, IMD, smoking status, duration of type 2 diabetes, HbA1c, number of type 2 diabetes medications, overall physical activity, accelerometer wear time and total energy intake, each 0.5g/kg of protein intake was associated with an 18.9% (95% CI: 2.3, 35.5) higher SPPB score, 22.7% (1.1, 44.3) more repetitions in STS‐60, 21.1% (4.5, 37.7) faster gait speed and 33.2% (16.9, 49.5) lower chair rise time. The interpretation remained similar after further adjustment for BMI (ESM Table S4). There were no associations with handgrip strength or any sex, HbA1c, age or BMI interactions.

**FIGURE 2:**
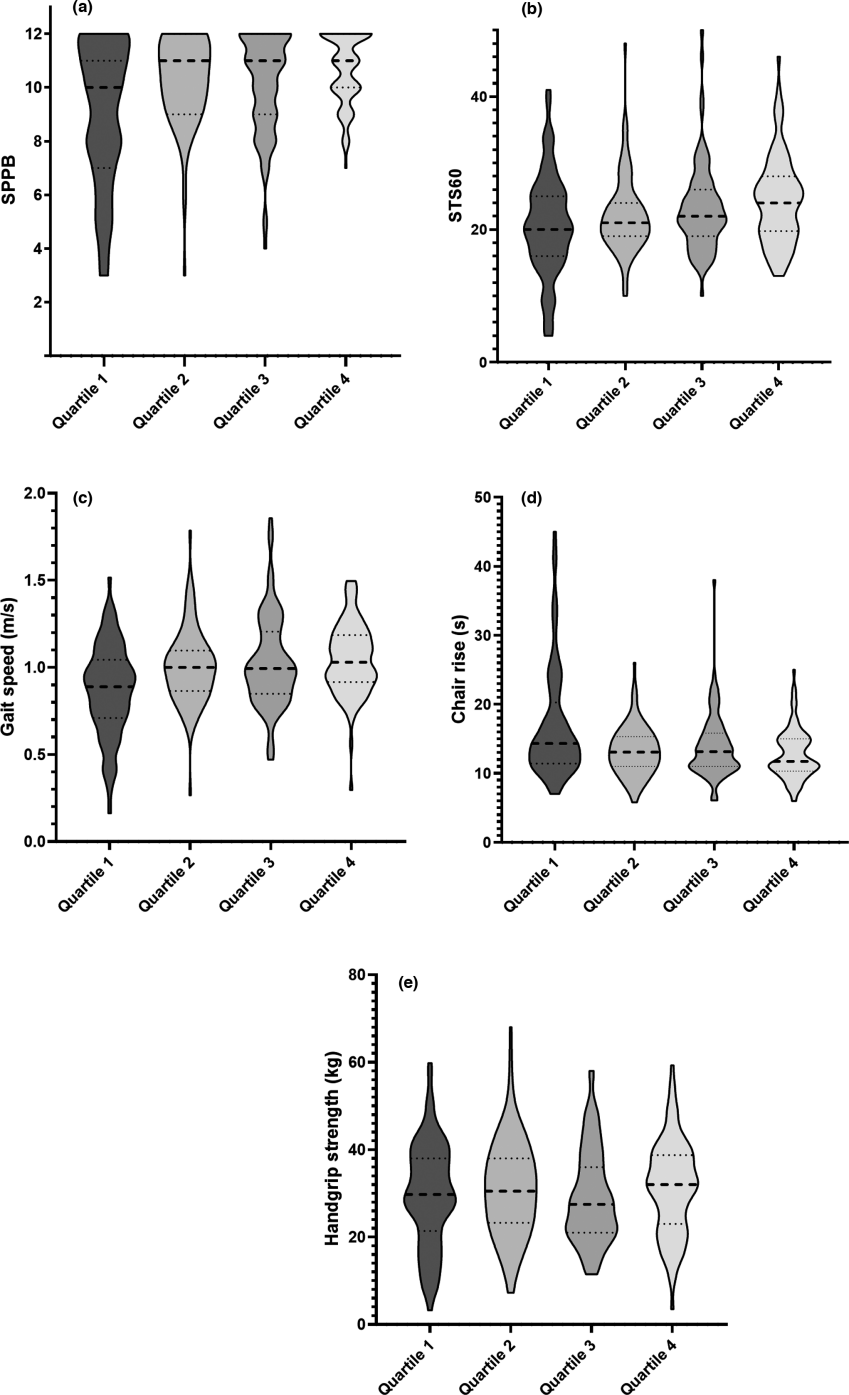
Violin plots demonstrating the quartiles of relative protein intake (g/kg/day) with SPPB score (a), STS60 (b), gait speed (c), chair time (5STS) (d) and handgrip strength (e). Quartile cut‐points for relative protein intake were 0.68, 0.84 and 0.94 g kg^−1^ day^−1^. The width of each curve corresponds with the approximate frequency of data points in each region. Median = thick dashed line and interquartile ranges (thin dotted line)

Figure 2 and ESM Table S5, illustrate the associations between quartiles of relative protein intake (reported as median (IQR) or estimated marginal mean (95% CI), respectively) and included outcomes, with dose response associations confirmed for SPPB, STS60, gait speed and chair rise time. When examining the SPPB score, compared with those in the lower quartile (median, IQR = 0.56 (0.47, 0.63) g kg^−1^ day^−1^), those in the upper quartile (median = 1.21 (1.12, 1.31) min) displayed a higher score 11.12 (10.24, 12.00) vs. 9.82 (9.02, 10.68), *p* for trend = 0.035. Similarly, those in the highest quartile (vs. the lowest quartile), performed more STS60 repetitions (26.27 (23.29, 29.62) vs. 20.23 (17.77, 23.03), *p* for trend = 0.022), displayed a faster gait speed (1.09 (0.96, 1.22) vs. 0.87 (0.74, 1.01), *p* for trend = 0.025) and a lower chair rise time (9.87 (7.07, 12.67) vs. 15.60 (12.68, 18.52), *p* for trend = 0.020).

## 4 DISCUSSION

Our analysis suggests that protein intake (reported as g kg^−1^ day^−1^ or g/day) is associated with markers of physical function (SPPB, STS‐60, gait speed (4mGS) and chair rise time (5STS)) in those with type 2 diabetes, independent of important confounders, including total energy intake, HbA1c, BMI, duration of diabetes and total physical activity. When examined as quartiles, the results confirm a dose‐response relationship. The findings were also consistent across sex, age, BMI and HbA1c categories. Over a third of individuals failed to meet the UK RNI for protein (≥0.75 g kg^−1^ day^−1^)^9^ and 87% did not reach European thresholds for older adults with chronic conditions (≥1.2 g kg^−1^ day^−1^).^10^ These population‐wide recommendations for protein intake may, therefore, not necessarily reflect the altered requirements for those with type 2 diabetes.

The results of our analysis extend previous findings in older adults, such as an epidemiological meta‐analysis where protein intake was inversely associated with frailty.^20^ More specifically, a 32% lower risk of frailty has been reported for every 20% increase in protein intake in older women.^21^ One of the few previous studies reporting on protein intake and gait speed also demonstrated positive associations in women with higher protein intake (≥1.2 g/kg body weight).^22^ Conversely, Nygard et al. found no associations between protein intake and physical performance in older adults.^23^ The discrepancy may be explained by the levels of protein intake and the choice of outcome.

The ceiling effect in SPPB can reduce the possibility of detecting associations between protein intake and physical function. In Nygard et al. 48.9% of the participants reached the top score of 12 points (vs. 26.9% in our cohort) and relative protein intake was 1.1 g kg^−1^ day^−1^ (vs. 0.88 g kg^−1^ day^−1^ in our cohort).^23^ Our findings are consistent with previous studies indicating no cross‐sectional association between protein intake and grip strength^24^, ^25^, ^26^ but an association with a composite score of function.^24^ The lack of associations may be driven by the measurement properties of handgrip strength. Although it works reasonably as a population level marker of function and strength, and correlates with whole body muscle strength,^27^ the correlation between handgrip strength and limb strength/function is negligible.^28^ Therefore, it will introduce regression dilution into the models, so although it demonstrated a positive association (albeit small), it is less likely to be significant than the other more precise (sensitive) measures (e.g. gait speed). However, higher intakes of protein have been shown to be protective against loss of grip strength, suggesting it may still be an important marker when examining the maintenance of physical function.^26^

Dietary protein is crucial to maintain body homeostasis and function.^29^ In those with type 2 diabetes, the catabolic effect of insulin deficiency, coupled with intra‐myocellular lipid accumulation, means that insulin is unable to reduce muscle protein breakdown in the fed state, ultimately creating a negative muscle protein balance.^30^ The reduced ability to mount an anabolic response to protein feeding and to blunt muscle protein breakdown in the fasted state means that those with type 2 diabetes are at an even greater risk for impairments in physical function. This is exemplified by the average gait speed of our cohort (1.0 m/s), which equates to normal gait speed for an 80–89 year old – around 20 years older than the average age.^31^

Our findings suggest that the majority of those with type 2 diabetes are not achieving recommendations to maintain MPS.^10^ Our findings mirror those reported by Morris et al.,^32^ who carried out secondary analyses in an older (mean age 72 years), overweight (mean BMI = 28.3 kg/m^2^), UK cohort (type 2 diabetes status not reported). They found that 35% failed to meet the current UK RNI for protein and fewer than 15% met the ESPEN age‐specific recommendation. Current guidance for people with type 2 diabetes does not differ from those recommended for the general UK population, despite the presence of protein anabolic resistance.^33^ Opinion articles and consensus statements have suggested that older people should be encouraged to consume greater quantities of protein than the current recommendations.^34^ The importance of protein intake has also been demonstrated in those with type 2 diabetes, where a 3‐year follow‐up study showed adequate protein intake (>1.0 g kg^−1^ day^−1^) slowed down functional capacity decline in older women (vs. those without type 2 diabetes).^35^ Similarly, adults with type 2 diabetes (average age = 59 years) who did not meet US protein recommendations (≥0.8 g kg^−1^ day^−1^)^36^ demonstrated a greater number of physical limitations, with more than 50% reporting limitations in activities fundamental to daily living.^37^

The key objective for people with impaired physical function and type 2 diabetes is to prevent deterioration and improve functional status from a pre‐disability (impaired function) state. Optimising diet with adequate protein intake combined with exercise programmes, including aerobic and resistance training, has the potential to improve physical function and reduce the risks of sarcopenia and frailty via synergistic effects in improving muscle function and muscle mass.^38^ The MID‐Frail study showed significant beneficial effects of a multimodal intervention (resistance exercise, diabetes and nutritional education) on functional status in frail and pre‐frail older adults with type 2 diabetes (aged > 70 years).^39^ Whether a protein supplementation or augmentation approach attenuates the decline in lean body mass and improves physical function in younger adults with type 2 diabetes remains unclear. As a result, intervention studies assessing the possible synergistic effect of a multicomponent intervention (exercise plus protein optimisation) on muscle function in type 2 diabetes are warranted.

Our sample is broadly representative of our larger cohort of people with type 2 diabetes and when compared with a population level cohort including those with type 2 diabetes (*n* = 74,222) derived from the Clinical Practice Research Datalink (CPRD) in the United Kingdom, the average BMI (30.6 kg/m^2^ (CODEC) vs. 30.1 kg/m^2^ (CPRD)), HbA1c (6.9% (CODEC) vs. 6.9% (CPRD)) and age (65 (CODEC vs. 67.7 (CPRD)) were broadly similar.^40^ However, our study does have limitations. Due to the cross‐sectional nature of this study, causality cannot be established. The relationship between protein intake and physical function is also complicated by the possibility of reverse causality. For example, loss of appetite (a feature of poor physical function and frailty) could lead to lower protein intake. Additionally, as is the case with many observational studies, the collection of diet intake was self‐reported. As a result, recall bias may exist, leading to potential misreporting of diet data and an under/overestimation of intake. The diet diaries were accompanied by clear instructions and prompting to improve accuracy of participant recording. We considered the potential issues arising from fluctuation in dietary intake over the course of a week by accounting for both weekday and weekends in our analysis. In addition, we did not investigate the relationship between the meal distribution of protein intake and the quality of dietary protein, both of which have been previously associated with improved physical function.^41^

We conclude that protein intake is associated with physical function outcomes in those with type 2 diabetes, across age, sex, HbA1c and BMI categories, even after consideration of total energy intake, BMI, HbA1c and overall physical activity. Given that a significant number of individuals were below the UK RNI and European guidelines for older adults with chronic conditions, protein intake may be a modifiable factor of importance for people with a low protein intake and type 2 diabetes.

## CONFLICT OF INTEREST

The authors declare no conflicts of interest.

## Supporting information

Supplementary Material
