# Supplementary material for: Relative protein intake and associations with markers of physical function in those with type 2 diabetes
Source: Diabet Med. 2022 Apr 21;39(8):e14851. doi: 10.1111/dme.14851 (PMC9546206; doi:10.1111/dme.14851)
Supplement: Supplementary file 1 — Supplementary Material [file DME-39-0-s001.docx]

Electronic Supplementary Material Table 1. Inclusion and exclusion criteria

| Inclusion | Exclusion |
| --- | --- |
| - Participant is willing and able to give informed consent for participation in the study | - Participant is unwilling or unable to give informed consent |
| - Established type 2 diabetes (>6months since diagnosis) | - Anyone <18 years of age and >75 years of age |
| - Male or Female | - HbA1c above 86mmol/mol (10%) |
| - Aged 18-75 years inclusive | - BMI > 45kg/m² |
| - BMI ≤ 45kg/m² | - A regular cannabis user i.e. weekly use |
| - No known sleep disorders except OSA | - Have a terminal illness |
| - HbA1c up to and below 10% (86mmol/mol) | - A known sleep disorder that is not OSA |
| - On any glucose-lowering therapy or lifestyle modification for management of T1DM | - Regular use (≥ weekly) of the following medications;   - -wakefulness promoting agents Modafinil, Amphetamine derivatives, Methylphenidate   - sedatives including benzodiazepines, Z-drugs (zopiclone, zolpidem & zaleplon)   - - Melatonin, including Circadin and melatonin analogues   - - Clonazepam and other drugs for nocturnal movement disorders |
| - Good command of the English language |  |

Electronic Supplementary Table 2. Comparison of those included vs. remainder of CODEC cohort

|  | *Included participants*  *n=413* | *Remainder of CODEC cohort*  *n=577* | *p for difference* |
| --- | --- | --- | --- |
| *Demographic variables* | | | |
| Age (years) | 65 ± 7.7 | 62.9 ± 8.7 | <0.001 |
| Sex (female) | 137 [33.2] | 197 [34.1] | 0.483 |
| Ethnicity (white European) | 380 [92.0] | 446 [77.3] | <0.001 |
| Current smokers | 18 [4.4] | 33 [5.7] | 0.799 |
| Index of multiple deprivation rank | 19828.6 ± 8973.5 | 19855.3 ± 9653.6 | 0.813 |
| *Anthropometric variables* | | | |
| BMI (kg/m^2^) | 30.6 ± 5.1 | 31.4 ± 5.0 | 0.426 |
| Weight (kg) | 89.4 ± 19.0 | 90.5 ± 17.4 | 0.359 |
| *Cardio-metabolic variables* | |  |  |
| HbA1c (mmol/mol) | 55 ± 4 | 54 ± 4 | 0.066 |
| HbA1c (%) | 6.9 ± 1.4 | 7.1 ± 1.1 |  |
| Duration of type 2 diabetes (years) | 10.8 ± 7.4 | 10.8 ± 7.8 | 0.970 |
| *Medication*  Biguanide  Sulphonylurea  GLP-1RA  Insulin  DPP-4i  SGLT2i  Thiazolidinedione | 264 [63.9]  72 [17.4]  15 [3.6]  75 [18.2]  58 [14.0]  32 [7.7]  6 [1.5] | 399 [69.1]  116 [20.1]  39 [6.8]  154 [26.7]  74 [12.8]  55 [9.5]  8 [1.4] | 0.022  0.221  0.027  0.001  0.666  0.429  0.961 |
| *Device measured physical activity* | | | |
| Number of days | 6.9 ± 0.3 | 6.9 ± 0.4 | 0.572 |
| Total physical activity (m*g*)* | 21.6 ± 7.1 | 21.9 ± 7.0 | 0.951 |
| *Physical function* | | | |
| SPPB | 11 (10, 12) | 11 (9, 12) | 0.924 |
| Number achieving an SPPB score of <10 | 131 [31.7] | 176 [30.5] | 0.227 |
| STS-60 | 22.6 ± 7.0 | 22.6 ± 6.8 | 0.932 |
| 4mGS (m/s) | 1.0 ± 0.2 | 0.9 ± 0.6 | 0.202 |
| 5STS | 14.2 ± 5.7 | 14.3 ± 6.0 | 0.444 |
| Handgrip strength | 30.3 ± 10.9 | 30.0 ± 10.4 | 0.691 |
| Data presented as median (interquartile range), number [column percentage] or mean±SD  GLP-1RA - Glucagon-like peptide-1 receptor agonist  DPP-4i - Dipeptidyl peptidase-4 inhibitor  SGLT2i - Sodium-glucose transport protein 2 inhibitor  SPPB – Short Physical Performance Battery  STS – sit to stand  4mGS – 4m gait speed  * This value represents ENMO (Euclidean norm minus 1g) [16] | | | |

Electronic Supplementary Table 3. Associations between relative protein intake (per 0.5g/kg/day) and functional assessments.

|  | OR^┼^ | 95% CI | p-value | p-value for sex interaction | p-value for age interaction | p-value for HbA1c interaction |
| --- | --- | --- | --- | --- | --- | --- |
| SPPB | 1.189 | 1.023, 1.355 | 0.024 | 0.870 | 0.329 | 0.871 |
| STS-60 | 1.227 | 1.011, 1.443 | 0.047 | 0.367 | 0.511 | 0.632 |
| 4mGS (m/s) | 1.211 | 1.045, 1.377 | 0.011 | 0.529 | 0.158 | 0.229 |
| 5STS | 0.668 | 0.505, 0.831 | 0.045 | 0.395 | 0.082 | 0.954 |
| Handgrip strength (kg) | 1.052 | 0.937, 1.167 | 0.781 | 0.966 | 0.055 | 0.667 |
| Adjusted for age, sex, ethnicity, smoking status, socio-economic status, total energy intake (minus protein content), duration of type 2 diabetes, HbA1c, number of diabetes medications, accelerometer wear time and overall physical activity | | | | | | |

^┼^ Values represent the fold change and confidence interval 95% (CI) for each 0.5g/kg/day change in protein intake

Electronic Supplementary Table 4. Associations between absolute protein intake (per 25g/day) and functional assessments.

|  | OR^┼^ | 95% CI | p-value | p-value for BMI interaction |
| --- | --- | --- | --- | --- |
| SPPB | 1.107 | 1.013, 1.201 | 0.042 | 0.594 |
| STS-60 | 1.096 | 1.018, 1.174 | 0.035 | 0.880 |
| 4mGS (m/s) | 1.122 | 1.026, 1.219 | 0.025 | 0.180 |
| 5STS | 0.841 | 0.738, 0.944 | 0.029 | 0.135 |
| Handgrip strength (kg) | 1.054 | 0.880, 1.228 | 0.698 | 0.937 |
| Adjusted for age, sex, ethnicity, smoking status, socio-economic status, total energy intake (minus protein content), duration of type 2 diabetes, HbA1c, number of diabetes medications, BMI, accelerometer wear time and overall physical activity | | | | |

^┼^ Values represent the fold change and confidence interval 95% (CI) for each 25g/day change in protein intake

|  | Relative protein intake (g/kg/day)  Mean (95% CI) | | | | |
| --- | --- | --- | --- | --- | --- |
|  | Quartile | | | | |
|  | 1 | 2 | 3 | 4 | |
| SPPB score | 9.21  (7.85, 10.80) | 10.35  (9.13, 11.72) | 10.55  (9.32, 11.82) | 11.32  (10.44, 12.00) | |
| P for trend | 0.035 | | | | |
| STS60 | 20.21  (16.26, 25.2) | 23.74  (19.88, 28.34) | 24.33  (20.66, 28.065 | 29.18  (23.49, 36.24) | |
| P for trend | 0.022 | | | | |
| Gait speed (m/s) | 0.83  (0.70, 0.98) | 0.97  (0.84, 1.12) | 1.02  (0.89, 1.17) | 1.08  (0.90, 1.30) | |
| P for trend | 0.025 | | | | |
| Chair stand (s) | 14.62  (10.79, 19.82) | 10.90  (8.52, 13.94) | 9.95  (7.65, 12.94) | 8.44  (6.15, 11.58) | |
| P for trend | 0.020 | | | | |
| HGS (kg) | 23.75  (19.72, 28.60) | 24.66  (21.25, 28.63) | 23.50  (20.28, 27.22) | | 25.81  (21.25, 31.36) |
| P for trend | 0.627 | | | | |
| Adjusted for age, sex, ethnicity, smoking status, socio-economic status, total energy intake (minus protein content), duration of type 2 diabetes, HbA1c, number of diabetes medications, accelerometer wear time and overall physical activity | | | | | |

Electronic Supplementary Table 5. Quartiles of relative protein intake (g/kg/day) with markers of physical function.
